# Unlocking the Unique Potential of *Thymus pannonicus*: Exploring the Efficacy of Supercritical CO_2_ Extraction, with and Without Pre-Treatments

**DOI:** 10.3390/plants13243457

**Published:** 2024-12-10

**Authors:** Siniša Simić, Senka Vidović, Stela Jokić, Nataša Milić, Krunoslav Aladić, Zoran Maksimović, Jovana Drljača Lero, Aleksandra Gavarić

**Affiliations:** 1 Faculty of Technology Novi Sad, University of Novi Sad, Bulevar cara Lazara 1, 21000 Novi Sad, Serbia; sinisa.simic@uns.ac.rs (S.S.); senka.vidovic@uns.ac.rs (S.V.); 2 Faculty of Food Technology Osijek, Josip Juraj Strossmayer University of Osijek, Franje Kuhača 18, 31000 Osijek, Croatia; stela.jokic@ptfos.hr (S.J.); k2aladic@gmail.com (K.A.); 3 Faculty of Medicine, Department of Pharmacy, University of Novi Sad, Hajduk Veljkova 3, 21000 Novi Sad, Serbia; natasa.milic@mf.uns.ac.rs (N.M.); jovana.drljaca-lero@mf.uns.ac.rs (J.D.L.); 4 Faculty of Pharmacy, University of Belgrade, Vojvode Stepe 450, 11221 Belgrade, Serbia; zoran.maksimovic@pharmacy.bg.ac.rs

**Keywords:** *Thymus pannonicus*, supercritical carbon dioxide extraction, enzymatic pre-treatment, microwave pre-treatment, gas chromatography

## Abstract

Since ancient times, many plant species within the *Thymus* genus have been used due to their numerous health benefits, such as antimicrobial, anti-inflammatory, antiseptic, or diuretic activity. While many of the species within this genus were well known and described, *Thymus pannonicus* All. or Pannonian thyme remains relatively unexplored despite its unique chemical composition and activity. *T. pannonicus* is a small shrub, spread over central and eastern Europe, with a diverse, location-dependent chemical composition. Within this study, the citral chemotype of *T. pannonicus* was used, which up to our knowledge was found and described only in Northern Serbia. Therefore, in order to explore the composition of *T. pannonicus* low-polar extracts and to present a preliminary insight into their bioactivity, supercritical carbon dioxide (ScCO_2_) extraction was applied (with and without pre-treatments), followed by GC-MS analysis. Different ScCO_2_ extraction pressures were tested (100, 150, 200, 250, and 300 bar) in order to determine the most suitable conditions for the extraction of *T. pannonicus*’s low-polar fraction. Additionally, in order to maximize the yield of the extraction, two types of ScCO_2_ extraction pre-treatments were investigated (enzymatic and microwave). The highest extraction yield obtained from untreated plant material was 3.01% (*w*/*w*), and it was obtained at conditions of 150 bar and 40 °C, while the 4% (*v*/*w*) enzymatic pre-treatment, with the same conditions, provided a yield of 3.89%. For all of the obtained extracts, the GC-MS analysis showed that oxygenated monoterpenes and sesquiterpenes were the two most dominant groups with principal bioactive compounds such as (E)-citral (18.95–38.17%), (Z)-citral (6.68–14.66%), β-bisabolene (8.2–14.4%), and nerol (6.08–9.67%). The extracts that exhibited the highest concentration of principal bioactive compounds ((Z)-citral, and (E)-citral) were further analyzed for anticancer potential, using short- and long-term cell viability observations on liver cancer cells.

## 1. Introduction

Consisting of approximately 250 different species and varieties distributed over Europe, Asia, and Africa, the genus *Thymus* is one of the most important and taxonomically complex genera within the Lamiaceae family [1]. Within the *Thymus* genus, several species have been used, since ancient times, for the production of essential oils (EOs), mainly because of their numerous health-beneficial effects [2]. It has been proven that the most common and major EO components occurring within the *Thymus* genus are phenol monoterpene derivatives of cymene, thymol, and carvacrol. Specifically, species like *T. vulgaris* (common thyme) and *T. zygis* (Spanish thyme) are of high commercial importance due to their high contents of thymol [3]. Thanks to the number of bioactive constituents, plant species within this genus are considered important medicinal plants. Therefore, many studies investigated their different medicinal effects such as antimicrobial, anti-inflammatory, antiseptic, antirheumatic, carminative, diuretic, and expectorant potentials.

*Thymus pannonicus* All. (Hungarian or Pannonian thyme) is a relatively unexplored species; it is a small shrub, 10–25 cm high, spread over central and eastern Europe. In the scientific literature, a couple of different chemotypes have been recorded, such as the Ukrainian chemotype (33.7–70.2% geraniol and 65.8% carvacrol), the Bosnian chemotype (13.7% thymol), or the German chemotype, which contains high levels of geranyl acetate [4]. For this study, a characteristic *T. pannonicus* citral chemotype was used. The *T. pannonicus* citral chemotype is only found and described in Northern Serbia, and contrary to all other described chemotypes, it contains geranial and neral (41.4 and 29.6%, respectively) as major components of its essential oil [5]. The essential oil of *T. pannonicus* has a characteristic lemon-like scent due to the high contents of geranial and neral, which when combined create citral. Thanks to the pleasant aroma, the dried herb of *T. pannonicus* has traditionally been used for the preparation of herbal tea drinks or the aromatization of food. Also, it has been used as a remedy for coughs and respiratory problems, as well as for the treatment of gastrointestinal disorders. Although there are just a couple of studies that investigated the medicinal activity of this citral chemotype of *T. pannonicus*, the available results stated that the essential oil of this plant exhibited potent antibacterial activity [5]. Additionally, it was reported that deodorized water extracts of *T. pannonicus*, obtained as a residual product in the process of essential oil production, exhibited potential cytotoxic and antiproliferative activities [6].

Within this study, supercritical carbon dioxide (ScCO_2_) extraction was applied for the isolation and preparation of a *T. pannonicus* low-polar fraction. On the contrary, the traditional extraction techniques like Soxhlet extraction, which requires high temperatures, usage of toxic organic solvents, and prolonged extraction time, the ScCO_2_ extraction has none of the aforementioned negative characteristics. This means that isolated low-polar fractions, due to the low process temperatures, preserve their high-quality chemical composition (higher quality as thermal degradation is avoided), and there is no trace of unused solvent (including organic ones), which enables direct use of the produced isolate. The ScCO_2_ extraction uses inert gas, carbon dioxide, in its supercritical state, which means that the process of extraction is conducted at a temperature higher than 31.1 °C and a pressure higher than 73.8 bar. While in a supercritical state, CO_2_ obtains the characteristics of both a gas and liquid, with high liquid-like density and high diffusivity similar to gases. These characteristics, in addition to CO_2_ nontoxicity, make it an excellent solvent for low-polar and lipophilic compound extraction from various plant matrices. Additionally, it was proved that the pressure of extraction influences the CO_2_ density and, therefore, directly impacts the extraction’s yield and selectivity [7].

In order to improve the efficiency of the conventional supercritical extraction procedure, and at the same time to stay in line with the green chemistry concept, different green pre-treatments of the plant material were conducted to potentially increase the efficiency and the yield of the isolated bioactive components. Many recent studies described the usage of different types of pre-treatments such as mechanical, enzymatic, or chemical which are designed to disrupt the cellular structure of the plant material and therefore increase the extraction yield. A study conducted by Meyer et al. in 2012 [8] showed that flaking rapeseed in a roller mill increased the extraction yield 3.5 times, in comparison to the other pre-treatment techniques, while Crampon et al. 2013 [9] demonstrated the influence of different drying pre-treatments. In accordance with our previous research (Vladić et al. 2021 [10]), within this study, enzymatic and microwave pre-treatments were applied to raw *T. pannonicus* plant material to increase both the yield of the extraction as well as the recovery of the principal bioactive compounds.

In addition, according to the available literature, a significant number of papers reflect on the promising cytotoxic effects of many Thymus species on cancer cell lines [11]. However, currently, no studies are focusing on non-polar *T. pannonicus* extracts and their beneficial effect as a potential anticancer agent. Therefore, within this study, preliminary assessment of the potential anticancer activity of *T. pannonicus* low-polar extracts on rat hepatoma cell lines was performed. Hence, this is the first report on such investigations to broaden the medical significance of this species and represents a preliminary basis for advanced analysis of the molecular mechanisms involved in their effects.

Considering all that was mentioned above, the present study focused on two main process aspects in order to obtain the high-quality *T. pannonicus* fractions/low-polar extracts applying the green approach: (a) analysis of the impact of the process pressure (as a dominant process parameter) on the composition of the isolated low lipid fraction and (b) the coupling of the ScCO_2_ extraction process with the microwave and enzymatic pre-treatments to achieve improved isolation of the targeted and most valuable constituents. In order to assess the quality of the obtained extracts, a comprehensive GC-MS analysis was performed, and the contents of the identified non-polar compounds were compared. Additionally, for the extracts that exhibited the highest concentrations of the principal bioactive compounds ((Z)-citral and (E)-citral) for both untreated and pre-treated plant material, potential anticancer activity was assessed in terms of the short-term and long-term cytotoxic activity on rat hepatoma cells lines (H4IIE).

## 2. Results and Discussion

### 2.1. ScCO_2_ Extraction Yield

The ScCO_2_ extraction of *T. pannonicus* has been conducted with five different extraction pressures (100, 150, 200, 250, and 300 bar) and a fixed temperature (40 °C) and CO_2_ flow rate (0.27 kg/h). In addition, two different extraction pre-treatments, enzymatic (ENZ1 and ENZ2) and microwave (MW), were applied (Table 1).

**Figure 1:**
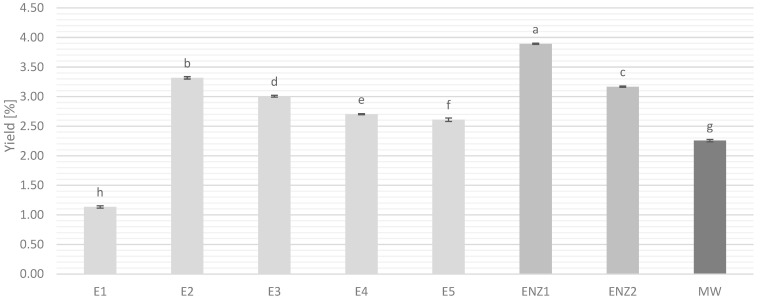
Yield of ScCO_2_ extraction for each extraction condition. Significant difference between samples (*p* < 0.05) is indicated by different letters.

Results with the low-pressure ScCO_2_ extraction (100 bar) provided a significantly lower yield of 1.14% (*w*/*w*) in comparison to all other runs conducted at higher pressures. This low yield obtained on the 100-bar extraction may be the consequence of the physical characteristics of CO_2_ at 100 bar and 40 °C, where its density (0.629 g/cm^3^) is not yet suitable for the extraction of most of *T. pannonicus* low-polar compounds. Immediately after the lowest pressure value, the second run—the ScCO_2_ extraction at 150 bar—provided the highest yield of 3.01% (*w*/*w*). After this point, an increase in the ScCO_2_ extraction pressure resulted in a slight but gradual drop in the extraction yield. These results indicate that the characteristics of the supercritical CO_2_ in terms of density and viscosity were the most suitable at 150 bar (Table 2, Figure 1). Additionally, a gradual drop in extraction yields also indicated that the primary low-polar components in *T. pannonicus* were of monoterpene and sesquiterpene structures rather than fatty acids and waxes, which was also confirmed by GC-MS analysis.

Similar results, in the extraction of some other herbs or oil-containing materials, were recorded by several studies. In Machmudah et al. 2007 [12], where the ScCO_2_ extraction of rosehip seeds was conducted with pressures ranging from 150 bar to 490 bar and a constant temperature of 40 °C, the highest yield was obtained at 150 bar, and a gradual drop in yields was seen with the increase up to 250 bar. In accordance, Morsy 2020 [13] reported that ScCO_2_ extraction of *Thymus vulgaris* with an extraction pressure of 16.7 MPa and temperature of 40 °C was the best method to obtain thymol-rich extracts. Additionally, Xiong K. and Chen Y. 2020 [14] reported that out of three tested extraction pressures (100, 150, and 200 bar), the ScCO_2_ extraction of tangerine peel essential oils provided the best results at 150 bar, and the kinetic model showed that optimal extraction conditions were 45 °C, 140 bar, and 147 min of extraction.

Because the extraction yield was highest at 150 bar, pre-treated *T. pannonicus* plant material was extracted at that pressure, and the effects of the pre-treatment were investigated. Keeping in mind the primary nature of low-polar compounds in the raw plant material, some losses during the pre-treatments were expected, and in accordance, the introduction of pre-treatments had a different influence on the extraction yield.

By applying the enzymatic pre-treatment, the disruption of the plant material integrity and enhanced extraction occurs due to enzyme hydrolysis of the plant cell wall structures [15]. Primarily, the plant cell walls consist of a complex network of cellulose, hemicellulose, pectin, and proteins which are essential for maintaining proper functioning of the cell. Different enzyme groups such as cellulase, carbohydrase, hemicellulase, and protease hydrolyze the components of the cell walls leading to increased cell wall permeability or disruption of the cell wall in its totality, which then facilitates the extraction of biocomponents [16]. The application of enzyme pre-treatments to extractions results in reduced extraction time, less solvent and energy consumption, and an increase in the extraction yield without degradation of targeted biomolecules; however, this procedure is demanding in terms of process parameters such as very specific temperature and pH requirements [17]. Considering a number of the studies reporting its effectiveness in cell wall disruption, a cellulolytic enzyme mixture Viscozyme (carbohydrase, arabanase, cellulase, β-glucanase, hemicellulase, and xylanase) was also used within this study for the pre-treatment of *T. pannonicus* plant material.

Application of 4% (*v*/*w*) cell-wall degrading enzyme (ENZ1) provided a yield of 3.89% which is a significant increase in the extraction yield of 14.65% (1.17 times) compared to untreated plant material. Vladić et al. 2021 [10] reported a similar increase in the ScCO_2_ extraction yield when oregano plant material was pre-treated with the same cell-wall degrading enzyme mixture at a concentration of 8%. The lower concentration of enzyme (2%) has not influenced the increase in the *T. pannonicus* extraction yield, and the result was just slightly lower than the extraction of untreated material at 150 bar. This slight drop in the extraction yield can be attributed to the losses during the process of pre-treating the material.

Contrary to the enzymatic pre-treatment, with the application of the microwave pre-treatment (MW), the plant material receives microwave irradiation directly through interactions with the electromagnetic field and the polar molecules such as water (moisture) within the matrix. As a result, heat is rapidly generated throughout the whole volume of the material, offering reduced processing time and less energy consumption. In addition, rapid delivery of the energy causes internal superheating of the material which subsequently disrupts both internal and external cell structures of the plant material which facilitates extraction procedures and recovery of the biomolecules [18,19].

Microwave pre-treatment showed a significantly lower yield in the extraction compared to both ENZ1 pre-treatment and extraction of untreated material. This drop in extraction yield is most likely the consequence of the chemical structure and polarity of the identified compounds, which were most likely extracted during the pre-treatment itself. Additionally, this also indicates that the power of MW pre-treatment was too strong which led to rapid degradation of the plant material’s cell walls and the extraction of targeted compounds. Regardless, although the yield of the extraction was decreased by the application of the MW pre-treatment, later GC-MS analysis showed that the MW extract exhibited high concentrations of principal bioactive compounds (highest content of (Z)-citral). Comparably, Da Porto et al. 2016 [20] reported that the application of MW pre-treatment increases the yield of an ScCO_2_ extraction of *M. oleifera* seeds up to a point of 1.5 min pre-treatment time and a power of 100 W. In conclusion, the results of this study indicate that acquiring the highest *T. pannonicus* ScCO_2_ extraction yield is achieved with a 4% enzymatic pre-treatment in combination with a pressure of 150 bar, while the MW pre-treatment provided an extract rich in bioactive compounds.

### 2.2. Gas Chromatography–Mass Spectrometry (GC-MS) Analysis

**Figure 2:**
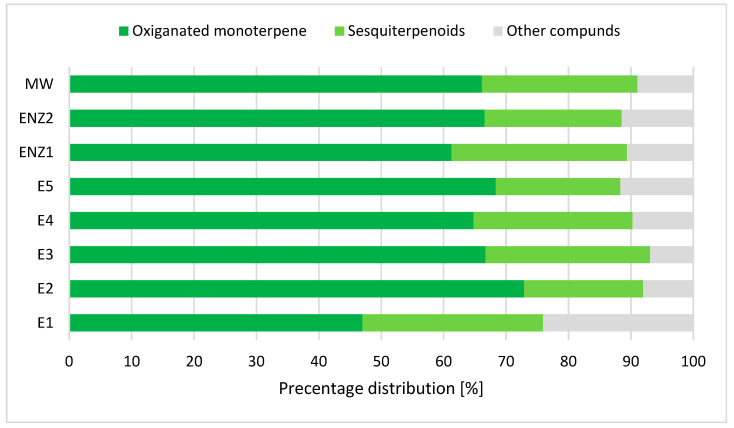
Percentage of the oxygenated monoterpene and sesquiterpenoids for all of the obtained *T. pannonicus* low-polar extracts [%].

The yield of ScCO_2_ extraction is by itself an important parameter and indicator of the successful extraction process because it provides first-hand information about the quality and characteristics of the investigated plant material. However, in order to have complete insight into the bioactivity of the obtained extract, a detailed analysis needs to be conducted with the purpose of determining principal bioactive components. Therefore, the GC-MS analysis was conducted for *T. pannonicus* fractions obtained for all extraction conditions. Additionally, the application of pre-treatments induced an increase in the extraction yield, but it is also important to determine whether the treatment impacted the bioactive components; therefore, the GC-MS analysis was conducted for all of the extracts obtained using pre-treated samples. The GC-MS analysis results showed a total of 38 detected components in the investigated samples, representing from 90.l6 to 96.2% of the extract, depending on the extraction conditions. Out of the identified compounds, two dominant groups emerged: oxygenated monoterpenes and sesquiterpenoids. Oxygenated monoterpenes accounted for 46.98% of the extract obtained at a pressure of 100 bar (E1) and up to 72.9% of the extract obtained at 150 bar (E2). In total, only the extract obtained at 100 bar had less than 50% oxygenated monoterpene. The second most dominant group of components, sesquiterpenoids, made up from 19.08 to 28.94% of the obtained extracts (Table 3; Figure 2).

Similar results were reported in the study by Maksimović et al. 2008 [5], which analyzed the essential oil of the same citral chemotype of *T. pannonicus*, where GC-MS analysis showed that 97.75% of the essential oil was composed of oxygenated monoterpenes. Maksimović et al. 2008 [5] also identified the presence of monoterpene hydrocarbons, sesquiterpenes, and oxygenated sesquiterpenes in much lower concentrations and characterized them as background components which is also in accordance with the results reported within this study. Contrary to this study, Maksimović et al. 2008 [5] reported that 71% of the oxygenated monoterpenes within the essential oil were geranial (41.42%) and neral (29.61%), which in combination create the lemon-scented citral. Within this study, however, it is proved that the extracts do not have geranial and neral in their composition but, rather, have their isomeric forms; that is, the extracts possess high concentrations of (Z)-citral (6.68–14.66%) and (E)-citral (18.95–38.17%). The reason for this difference was probably due to the different applied extraction techniques because the ScCO_2_ extraction was conducted at a mild temperature of 40 °C, while hydrodistillation required a temperature of about 100 °C, which could lead to the temperature transformation of certain compounds, as well degradation of the most heat-sensitive ones.

**Figure 3:**
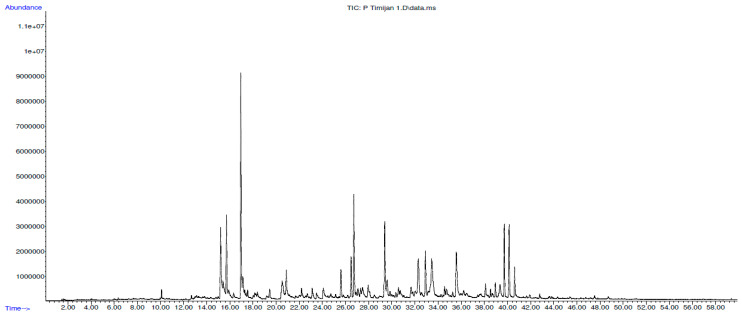
Total ion chromatogram (TIC) for the sample E1, with the *x*-axis representing time [min] and the *y*-axis representing signal intensity.

The GC-MS analysis of *T. pannonicus* ScCO_2_ extracts showed the presence of several distinct principal bioactive compounds (Figure 3). Noticeably, bioactive components that were in the highest content, with the variations depending on the extraction conditions, were the following: (E)-citral (18.95–38.17%), (Z)-citral (6.68–14.66%), β-bisabolene (8.2–14.4%), nerol (6.08–9.67%), and neryl acetate (2.15–4.57%), as well as neric acid, germacrene D, spathulenol, and α-cadinol, which were in slightly reduced concentrations. Up to now, there are several published studies which reported on the bioactivity of these compounds. Chaouki et al. 2009 [21] reported that citral treatment had a high antiproliferative effect on the MCF-7 breast cancer cell line. Additionally, Sheikh et al. 2017 [22] reported that citral treatment induced apoptosis in human colorectal HCT116 and HT29 cell lines. A recent study by Nabila et al. 2024 [23] reported that sesquiterpene β-bisabolene has potential antidiabetic activity, while nerol and neryl acetate exhibit antibacterial activity against several bacterial strains [24].

The results of the GC-MS analysis also showed that several distinct differences could be observed between extracts obtained from the untreated plant material at pressures from 150 bar to 300 bar and the extract obtained from the same material at 100 bar of pressure. Compared to other extracts, E1 had the lowest content of oxygenated monoterpenes (46.98%) and the highest content of sesquiterpenoids (29.94%). Additionally, E1 showed a distinctively high content of compounds that were present in low concentration in all other extracts, specifically bisabolol oxide A (5.88%), 1,2-benzendicarboxylic acid (4.8%), spathulenol (7.01%), and α-cadinol (5.62%). A common characteristic for all of these compounds is that their molecular weights are distributed in close proximity (188.11, 220.35, 222.37, and 238.37 g/mol for 1,2-benzendicarboxylic acid, spathulenol, α-cadinol, and bisabolol oxide, respectively), which can be the reason why the density of CO_2_ at 100 bar was the most suitable for their extraction. Additionally, the location of these compounds in specific parts of the plant material (surface layers) can also influence their better extraction with a lower ScCO_2_ extraction pressure.

The ScCO_2_ extraction conditions resulted in the highest content of oxygenated monoterpenes (72.9%) in the E2 extract, the one obtained at a pressure of 150 bar. Accordingly, the highest concentrations of (Z)-citral and (E)-citral in the extracts obtained without pre-treatment were recorded in the same E2 extract (14.66 and 38.17%, respectively). For the other bioactive compounds, the highest values were distributed among the extracts obtained with ScCO_2_ pressure conditions from 200 to 300 bar. Extract E3 had the highest concentration of neryl acetate (4.76%) and extract E5 had the highest concentration of nerol and neric acid (9.70 and 7.76%, respectively). The concentration of sesquiterpenes obtained with ScCO_2_ pressure conditions from 150 to 300 bar (E2–E5) was in the range from 19.08 to 26.35%, with the extract E3 having the highest content of germacrene D and β-bisabolene (3.78 and 13.37%, respectively).

Pre-treatments of *T. pannonicus* plant material led to high concentrations of total oxygenated monoterpenes in all the obtained extracts. Interestingly, pre-treatments that resulted in lower extraction yields (ENZ2 and MW) had a higher concentration of oxygenated monoterpenes compared to ENZ1 which increased the yield of ScCO_2_ extraction. Additionally, while the ENZ1 extract had a higher concentration of (E)-Citral (35.62%), both ENZ2 and MW had higher concentrations of (Z)-citral (11.99 and 14.82, respectively). Application of enzymatic pre-treatments resulted in extracts with the highest recorded concentration of the sesquiterpenoid compound β-bisabolene of 14.4% (ENZ1), while microwave pre-treatment resulted in the highest concentrations of (Z)-citral and germacrene D (14.82 and 4.10%, respectively). In addition, it is important to mention that the application of enzymatic pre-treatments resulted in the presence of acetic acid in the obtained extracts since the pre-treatment itself was conducted in an acetate buffer. This distribution in concentrations of bioactive compounds is a clear indication that with the change in ScCO_2_ extraction conditions the properties of the CO_2_ as a solvent change, and, therefore, the selectivity of the extraction changes. Additionally, these results also indicate that the application of plant material pre-treatments, in addition to the increase in the yield of the extraction, can alter the composition of low-polar compounds in ScCO_2_ extracts with the same extraction conditions.

### 2.3. Anticancer Potential

The cytotoxic and anticancer potential of EOs, extracts, and phytocompounds of different *Thymus* species have been explored, particularly using in vitro cellular models [11]. *Thymus* spp. has shown anticancer potential in lung adenocarcinoma, prostate cancer cells, breast cancer cells, ovarian cancer cells, and leukemia [25,26,27,28]. However, so far, no information regarding the antiproliferative effect of ScCO_2_ low-polar extracts of *T. pannonicus* was found in the literature.

In this study, rat hepatoma cells (H4IIE) were used to examine the effects of 0–300 μg/mL of a supercritical CO_2_ extract of *T. pannonicus*, with or without microwave pre-treatment, on short- and long-term cell viability. For this analysis, extracts E2 and MW were selected based primarily on the concentration of (Z)-citral and (E)-citral, as two of the principal bioactive components with reported anticancer activity. Both the E2 and MW extracts exhibited the highest concentration of (Z)-citral for raw and pre-treated plant material (14.66 and 14.82%, respectively). For the concentration of (E)-citral, E2 exhibited the highest concentration of 38.17%; MW had the second highest concentration of 35.59%. Although, in order to have a complete assessment, all of the extracts should be analyzed. This study serves as a preliminary investigation into the anticancer potential of the *T. pannonicus* low-polar extracts, as well as citral as a bioactive compound. Therefore, within this study, only extracts E2 and MW were analyzed for short-term and long-term cell viability, while the subsequent studies will aim to produce comprehensive research into the anticancer potential of *T. pannonicus* low-polar fractions.

**Figure 4:**
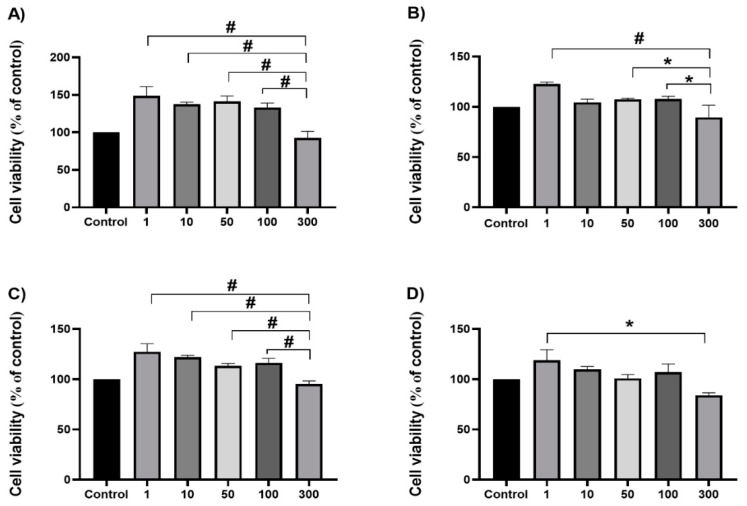
Effects of different concentrations of *T. Pannonicus* E2 (**A**,**B**) and MW (**C**,**D**) extracts, in the concentration range 0–300 µg/mL, on H4IIE short-term cell viability after 24 h and 48 h of treatment. Data are presented as mean ± S.D. * *p* < 0.05; # *p* < 0.001.

As a first approach method, the MTT assay was used to estimate the potential cytotoxicity of the extracts on the cancer cell line after 24 h and 48 h of treatment (Figure 4). After 24 h and 48 h of treatment, neither the E2 nor MW extract showed cytotoxic effects compared to control. On the contrary, a slight increase in cell viability was observed after incubation with low doses of the extracts. This increment in cell viability is not surprising and could be attributed to their antioxidant potential [29].

**Figure 5:**
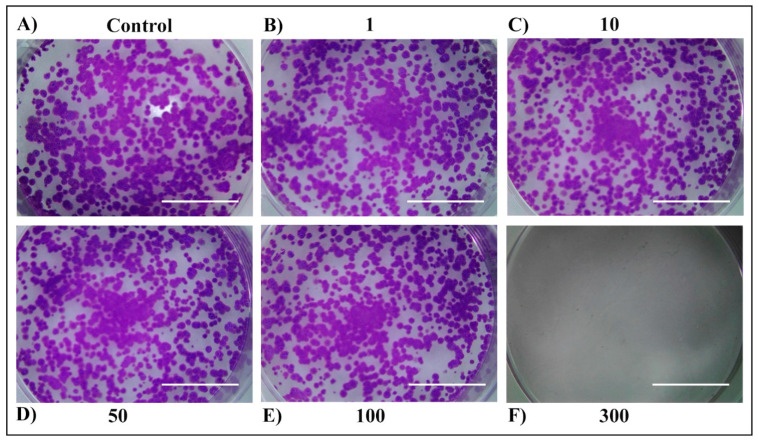
Long-term survival of H4IIE cells following the 24 h treatment with *T. Pannonicus* E2 extract in the concentration range 0–300 µg/mL. (**A**) Control. (**B**) Extract concentration 1 μg/mL. (**C**) Extract concentration 10 μg/mL. (**D**) Extract concentration 50 μg/mL. (**E**) Extract concentration 100 μg/mL. (**F**) Extract concentration 300 μg/mL. The scale bar represents 1 cm.

**Figure 6:**
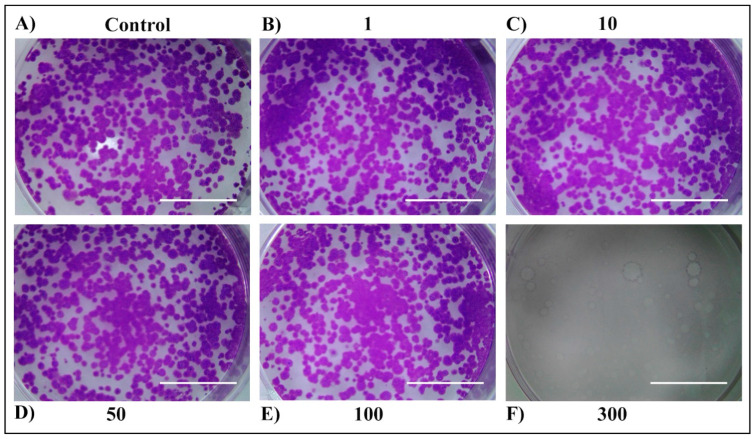
Long-term survival of H4IIE cells following the 48 h treatment with *T. Pannonicus* E2 extract in the concentration range 0–300 µg/mL. (**A**) Control. (**B**) Extract concentration 1 μg/mL. (**C**) Extract concentration 10 μg/mL. (**D**) Extract concentration 50 μg/mL. (**E**) Extract concentration 100 μg/mL. (**F**) Extract concentration 300 μg/mL. The scale bar represents 1 cm.

**Figure 7:**
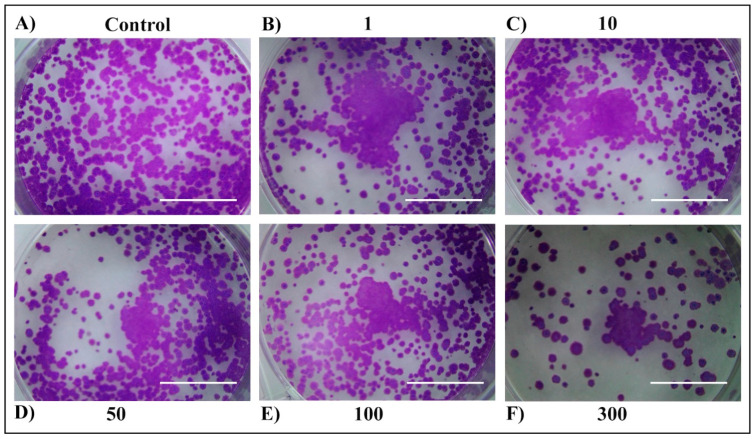
Long-term survival of H4IIE cells following the 24 h treatment with *T. Pannonicus* MW extract in the concentration range 0–300 µg/mL. (**A**) Control. (**B**) Extract concentration 1 μg/mL. (**C**) Extract concentration 10 μg/mL. (**D**) Extract concentration 50 μg/mL. (**E**) Extract concentration 100 μg/mL. (**F**) Extract concentration 300 μg/mL. The scale bar represents 1 cm.

**Figure 8:**
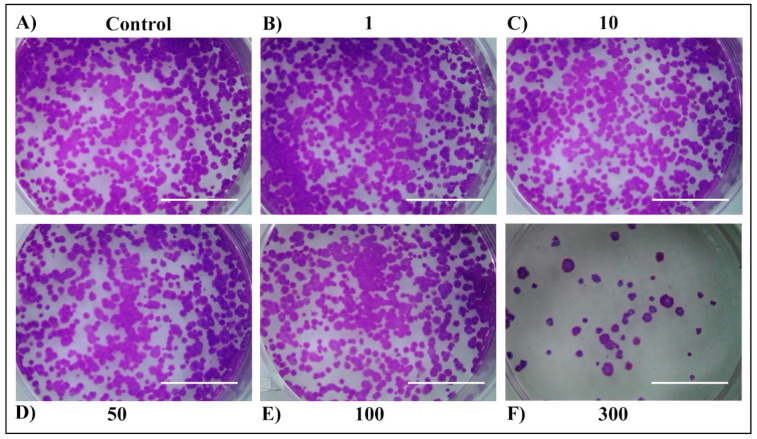
Long-term survival of H4IIE cells following the 48 h treatment with *T. Pannonicus* MW extract in the concentration range 0–300 µg/mL. (**A**) Control. (**B**) Extract concentration 1 μg/mL. (**C**) Extract concentration 10 μg/mL. (**D**) Extract concentration 50 μg/mL. (**E**) Extract concentration 100 μg/mL. (**F**) Extract concentration 300 μg/mL. The scale bar represents 1 cm.

For long-term cell survival observation, a colony-formation assay was performed (Figure 5, Figure 6, Figure 7 and Figure 8). First, no difference was observed between 24 h and 48 h of treatment within the same extract. Second, both extracts exerted a strong anticancer potential, but only when used at the concentration of 300 µg/mL. The third, stronger anticancer potential was observed in the E2 extract, in which none of the colonies could be found when treated with 300 µg/mL, compared to the MW extract, in which a small number of colonies remained. The higher anticancer potency of the E2 extract in comparison to the MW extract could be attributed to the better extraction yield and total terpene profile. This discrepancy between the short-term and long-term cell cytotoxicity could be due to the delayed effects of the extracts, which are mechanism dependent. The cytotoxic effect could arise by triggering different cell death pathways. Many genes are effective in apoptosis which is the most important cell death pathway [30]. Even though the mechanism of the observed anticancer potential of tested extracts in H4IIE cells is unknown, due to their low-polar nature, monoterpenes and sesquiterpenoids appear to increase the permeability of the cell membrane, leading to the generation of metabolites and enzyme leakage [31,32].

Furthermore, citral is the principal component in both extracts. Duerksen-Hughes et al. 1999 [33] established the genotoxic property of citral due to the p53 protein increase, resulting in DNA damage. Moreover, Souza et al. 2020 [34] indicated the significant genotoxic impact of citral in the human hepatocellular HepG2 cell line. Generally, the biological activity of plant extracts is related to their phytochemical composition. Nevertheless, the less present phytochemicals must be considered, since the extract’s compounds could show synergistic/additive effects [35,36]. Whether citral alone or in combination with other components of the ScCO_2_ extracts is responsible for the observed cytotoxicity against liver cancer cells remains to be established.

## 3. Materials and Methods

### 3.1. Plant Material and Chemicals

*T. pannonicus* whole plant material was provided from the personal cultivation of Prof. Dr Zoran Maksimović, Institute of Pharmacognosy, School of Pharmacy, University of Belgrade. The plant material was air dried, the content of moisture was 7.87%, and it was determined using a standard gravimetric procedure. After the drying, the herb was ground using an industrial scale mill to a mean particle size of 0.49 mm which was calculated using vibration sieve sets (CISA, Cedaceria, Spain).

Carbon dioxide for the ScCO_2_ extraction with a purity > 99.98% (*w*/*w*) was obtained from a local provider Messer, Novi Sad, Serbia. Viscozyme, a cellulolytic enzyme complex (carbohydrases, arabanase, cellulase, β-glucanase, hemicellulose, and xylanase) that was used for the enzymatic pre-treatment, was purchased from Sigma-Aldrich (St. Louis and Burlington, MA, USA).

### 3.2. Enzymatic Pre-Treatment of Plant Material

Enzymatic pre-treatment of *T. pannonicus* plant material was conducted with two different concentrations of the cellulolytic enzyme Viscozyme. The first enzymatic pre-treatment was conducted with 4% Viscozyme (*v*/*w*), while the concentration for the second pre-treatment was 2% (*v*/*w*). The percentage was calculated based on the dry weight of the plant material, and the samples were marked as ENZ1 and ENZ2 for the 4 and 2% Viscozyme concentration, respectively. In both samples, Viscozyme was dissolved in an acetate buffer solution (pH 4.6) and mixed with the *T. pannonicus* plant material. The ratio of plant material to buffer solution was 1:5 (*w*/*v*), and after mixing, the mixture was tempered at 45 °C for 60 min. After the tempering, the plant material was separated from the buffer solution using vacuum filtration and freeze-dried for 48 h. The dried material was used for the ScCO_2_ extraction.

### 3.3. Microwave Pre-Treatment of Plant Material

The second type of pre-treatment was the disruption of plant material cell structures using microwave irradiation. Firstly, plant material was mixed, in a round flask, with distilled water in a ratio of 1:5 (*w*/*v*) and placed in a modified commercial microwave oven (NN-E201W, Panasonic, Osaka, Japan). The microwave irradiation power was set to 800 W, and the time of pre-treatment was 2 min. The appropriate time and power for the pre-treatment were determined based on previous research [10]. After the pre-treatment, plant material was separated from the liquid phase using vacuum filtration and freeze-dried for 48 h (Alpha 1–2 LPlus, Christ, Osterode am Harz, Germany). The dried samples were used for the ScCO_2_ extraction.

### 3.4. Supercritical Carbon Dioxide Extraction

For the supercritical carbon dioxide (ScCO_2_) extraction, a laboratory-scale, high-pressure system (HPEP, NOVA-Swiss, Effretikon, Switzerland) was used. The main components of the ScCO_2_ extraction system were a CO_2_ gas container, high-pressure compressor (pressure range up to 1000 bar), extraction unit (200 mL volume; maximum working pressure of 700 bar), separation unit (200 mL volume; maximum working pressure of 250 bar), pressure control valves system, temperature control unit, and regulation valves.

The extraction for the untreated plant material was performed at five different pressures (100, 150, 200, 250, and 300 bar) at the constant extraction temperature of 40 °C and with an extraction time of 3 h. Because the yield of the extraction was highest for the conditions of 150 bar and 40 °C, the enzymatic- and microwave-pre-treated plant material was processed by supercritical CO_2_ only under those conditions. For all of the extractions, pressure in the separation unit was held at 150 bar, and the flow of ScCO_2_ was constant at 0.27 kg/h. For each extraction run, 30 g of ground and dried plant material was used, and each extraction was performed in triplicate. Obtained extracts were collected in plastic cuvettes, protected from direct sunlight, and stored at 4 °C for further analysis.

### 3.5. Gas Chromatography–Mass Spectrometry (GC-MS) Analysis

GC-MS analysis was performed using an Agilent Technologies (Palo Alto, CA, USA) gas chromatograph model 7890A coupled with a 5975C mass detector (Agilent Technologies). For the analysis, an HP-5MS capillary column was used (5% phenyl-methylpolysiloxane stationary phase, 30 m length, 0.25 mm diameter, and coating thickness of 0.25 μm). The carrier gas was helium at 1 mL/min, while the temperature of the injector was 250 °C. The oven temperature was programmed to be isothermal at 70 °C for 2 min, increasing from 70 to 200 °C at 3 °C/min, and held isothermally at 200 °C for 15 min. The split ratio was 1:20, and the ionisation voltage was 70 eV; the ion source temperature was 230 °C, and the temperature of the quadrupole was 150 °C. The scanning mass range was from 30 to 450 amu. The percentage of the components was calculated from the GC peak areas as mean values from duplicate GC-MS analysis of all the extracts.

For the solid phase microextraction of the *T. pannonicus* ScCO_2_ extracts, a DVB/CAR/PDMS fiber was chosen (grey fiber with an 80 μm film thickness, Supelco Co., Bellefonte, PA, USA). Before use, the fiber was activated at 250 °C, in accordance with the manufacturer’s instructions. After the conditioning procedure, the fibers were used to extract a 0.01 g *T. pannonicus* extract sample which was placed in the 20 mL glass vial and hermetically sealed with a stopper with a septum. The PAL system automatically sampled the peak vapors of the *T. pannonicus* extract for 40 min with periodic mixing. After collecting the surface vapors, the fibers were transferred to a GC-MS injector (250 °C) for thermal desorption for 7 min. The analysis started immediately after the injection of the fiber into the inlet of the device.

The identification of the compounds was conducted using Wiley9 (New York, NY, USA), Nist11 (Gaithersburg, MD, USA), and the Pest database within the ChemStation (OpenLab CDS 2.7) software by Agilent (Palo Alto, CA, USA). For all of the components, the Kovats index was calculated and compared with the specters from said databases.

### 3.6. Cell Culture

Rat hepatoma cells were obtained from the American Type Culture Collection (H4IIE cell line, ATCC^®^ CRL-1548TM, Manassas, VA, USA). The cells were cultivated as a monolayer culture in polystyrene flasks with MEM medium, supplemented with 10% (*v*/*v*) fetal bovine serum, 100 U/mL penicillin, 0.1 mg/mL streptomycin, and 2 mM glutamine. Further, the cells were grown in a 5% CO_2_ atmosphere at 37 °C in humidified conditions. After reaching the exponential phase of growth, the cells were subsequently subcultured twice a week at a 1:10 ratio using 0.05% trypsine/EDTA. All reagents were from Capricorn Scientific GmbH, Ebsdorfergrund, Germany.

### 3.7. Preparation of Extract Solutions for Cellular Assays

The ScCO_2_ extracts of *T. pannonicus* plant material obtained at 150 bar and 40 °C with (MW) or without microwave pre-treatment (E2) were further tested for cytotoxicity. These extracts were dissolved in dimethylsulfoxide (DMSO) and diluted in the MEM medium before the treatment. To avoid false cytotoxic effects, the percentage of DMSO did not exceed 0.1%. The cells were treated for 24 h and 48 h with concentrations ranging from 0 to 300 µg/mL of the tested extracts (marked as 1, 10, 50, 100, and 300). Control was treated with 0.1% DMSO.

### 3.8. MTT Assay

The short-term cell viability was determined using the MTT (3-(4,5-Dimethylthiazol-2-yl)-2,5-diphenyl tetrazolium bromide) assay as we described earlier [37]. Twenty-four hours after seeding 20,000 cells per well in 96-well plates, the culture medium was replaced by a fresh medium containing the prepared extract solutions with the desired concentration. Following the treatment for 24 h and 48 h, the viability of the H4IIE cells was determined by the ability of viable cells to produce formazan from MTT [38]. Briefly, the medium-containing extracts were discarded, 0.5 mg/mL of MTT (Sigma-Aldrich, USA) dissolved in MEM medium was added to each well, and the plates were incubated at 37 °C for 3 h. Cell lysis and dissolution of formazan crystals were accomplished by adding 0.04 M HCl/isopropanol. The absorbance was read on a microplate spectrophotometer (Multiscan MCC340, Labsystems, Huntsville, AL, USA) at 540/690 nm. Cell viability was calculated as the percentage of viable cells in comparison to untreated cells (control). The assay was repeated three times in quadruplicate.

### 3.9. Colony-Forming Assay

A colony-forming assay was performed to determine the long-term cytotoxicity of tested extracts, where the effects of 24 h and 48 h treatments on small cell numbers were followed for two weeks. Twenty-four hours after seeding 2000 cells per well in 6-well plates, the culture medium was replaced by a fresh medium containing the prepared extract solutions with the desired concentration. Following the treatment for 24 h and 48 h, the tested extracts solutions were discarded, and for the next 14 days, the cells were maintained in a fresh complete medium. Following the cell fixation with methanol, cells were dyed with 0.5% crystal violet solution (Sigma-Aldrich). Representative images for each group were taken using a stereomicroscope (OZR 564, KERN Optics, Balingen, Germany).

### 3.10. Statistical Analysis

Results from the MTT assay were analyzed using IBM SPSS version 26 (Chicago, IL, USA). Data are expressed as mean ± standard deviation (S.D.). The Shapiro–Wilk test was used to assess the normality of the data. To determine the existence of significant differences between the groups, data were analyzed with the one-way analysis of variance (ANOVA), followed by the Tukey test. A *p*-value lower than 0.05 was considered significant.

## 4. Conclusions

The results obtained within this study provided clearer insight into the characteristics of the *T. pannonicus* citral chemotype, in terms of the content of low-polar molecules, the presence of principal bioactive molecules, and its cytotoxic and anticancer potential. Additionally, green and ecologically safe ScCO_2_ extraction was applied at five different extraction pressures, with two different extraction pre-treatments (enzymatic and microwave), in order to determine the best approach for the utilization of this valuable plant material. The yield of ScCO_2_ extraction varied depending on the extraction pressure and the pre-treatment applied, with the highest achieved yield of untreated plant material obtained for the extract E2 (150 bar; 40 °C) which was 3.01% (*w*/*w*). Application of the enzymatic pre-treatment ENZ1 (4% Viscozyme, 150 bar, and 40 °C) led to an increase in the extraction yield and provided the total highest yield of 3.89% (*w*/*w*), which was an increase compared to E2 of 14.65%. GC-MS analysis was conducted for all of the extracts, and several principal bioactive components were detected, such as (E)-citral (18.95–38.17%), (Z)-citral (6.68–14.66%), nerol (6.08–9.67%), and β-bisabolene (8.2–14.4%). Additionally, GC-MS analysis showed that the extracts were mainly composed of oxygenated monoterpenes and sesquiterpenoides, with the highest content of oxygenated monoterpenes (72.9%) recorded in the extract E2. For the determination of the cytotoxic and anticancer potential, E2 and MW extracts were selected based on their concentrations of (Z)-citral and (E)-citral, and their anticancer potential was assessed for the short- and long-term cell viability of rat hepatoma cells (H4IIE). While the MTT assay that was used for estimation of the short-term cell viability did not show activity for the tested extracts, a colony-formation assay for long-term cell survival observation showed that extracts at the concentration of 300 μg/mL exhibit strong anticancer activity against H4IIE rat hepatoma cells.

## Figures and Tables

**Table 1:** Conditions of ScCO_2_ extractions for each *T. pannonicus* plant material sample.

Sample	Pre-Treatment	Pressure of Extraction [bar]	Extraction Temperature [°C]
E1	/	100	40
E2	/	150	40
E3	/	200	40
E4	/	250	40
E5	/	300	40
ENZ1	4% Viscozyme	150	40
ENZ2	2% Viscozyme	150	40
MW	800 W; 2 min	150	40

**Table 2:** Yield of ScCO_2_ extraction. Significant difference between samples (*p* < 0.05) is indicated by different letters.

Sample	Plant Material [g]	Yield [%]
E1	30.00	1.14 ± 0.02 ^h^
E2	30.00	3.32 ± 0.02 ^b^
E3	30.00	3.01 ± 0.02 ^d^
E4	30.00	2.70 ± 0.01 ^e^
E5	30.00	2.61 ± 0.03 ^f^
ENZ1	23.31	3.89 ± 0.01 ^a^
ENZ2	23.16	3.17 ± 0.01 ^c^
MW	23.83	2.26 ± 0.02 ^g^

**Table 3:** The GC/MS analysis of *T. pannonicus* extracts obtained by ScCO_2_ with different extraction conditions (E1–E5) and different applied pre-treatments (ENZ1, ENZ2, and MW). The obtained results were presented as % of total identified compounds, calculated as a relative peak area, and the principal components were highlighted.

Compound	Rt [min]	RI	E1	E2	E3	E4	E5	ENZ1	ENZ2	MW
*Oxygenated monoterpene*										
*Linalool*	10.086	1102	0.53	0.24	0.25	0.26	0.26	0.19	0.23	0.19
*Borneol*	12.674	1171	0.29	0.13	0.13	0.14	0.26	0.12	0.13	0.09
*4-Terpineol*	13.125	1182	0.21	0.07	0.07	0.08	-	-	0.08	0.07
***Nerol***	15.198	1232	9.67	8.43	8.36	8.26	9.7	7.28	8.32	6.08
***(Z)-Citral***	15.708	1245	6.68	14.66	12.42	12.35	11.32	8.86	11.99	14.82
*Geraniol*	16.297	1259	2.82	0.3	0.32	0.34	0.31	0.32	0.4	0.23
***(E)-Citral***	16.948	1274	18.95	38.17	35.17	32.15	31.12	35.62	32.19	35.59
*Carvacrol*	18.387	1306	0.27	0.32	0.25	0.32	0.36	0.22	0.53	0.13
*Geranic acid*	20.520	1360	1.84	0.46	0.09	0.43	1.23	-	6.86	-
***Neric acid***	20.653	1363	1.77	2.73	2.22	5.31	7.76	2.98	0.27	3.18
***Neryl acetate***	20.876	1368	2.15	4.57	4.76	3.03	3.41	3.17	3.74	2.87
*Geranyl acetate*	20.960	1370	0.74	1.03	0.94	0.74	1.41	0.92	-	1
*6-Methylhept-5-en-2-one*	6.342	990	0.1	0.06	-	-	-	-	0.05	0.04
*Car-3-en-2-one*	23.119		0.96	1.73	1.75	1.39	1.21	1.6	1.77	1.87
*Sesquiterpenoids*										
*β-Bourbonene*	21.700	1387	0.16	0.44	0.3	0.27	0.26	0.29	0.24	0.95
*Trans-β-Farnesene*	24.649	1460	0.19	-	0.06	-	-	-	-	0.07
*β-Selinene*	24.724	1462	0.47	-	0.43	0.32	-	0.64	0.3	1.14
***Germacrene D***	25.603	1483	2.08	3.44	3.78	3.25	2.27	3.64	2.14	4.1
*Trans-β-Ionone*	25.811	1488	0.12	0.14	0.15	0.16	0.12	0.13	0.28	0.26
*Bicyclogermacrene*	26.214	1497	0.32	0.36	0.4	0.32	0.36	0.34	0.12	0.39
***β-Bisabolene***	26.718	1510	8.2	7.45	13.37	12.31	7.89	14.4	10.38	11.61
*γ-Cadinene*	26.925	1516	0.32	0.31	0.54	0.35	0.26	0.48	0.33	0.31
*δ-Cadinene*	27.304	1526	0.71	0.72	0.91	0.69	0.41	0.73	0.79	0.67
*Salvial-4(14)-en-1-one*	29.985	1594	0.34	0.14	0.1	-	0.17	0.23	0.1	0.2
***Spathulenol***	29.390	1580	7.01	2.2	2.5	2.94	3.06	2.78	2.72	2.11
*Caryophyllene oxide*	29.572	1584	-	0.83	0.97	1	1.1	1.08	0.95	0.41
*Ledol*	30.355	1604	0.52	0.06	0.15	0.15	0.15	0.19	0.16	0.12
*Isospathulenol*	31.673	1641	1.14	0.4	0.25	0.55	0.52	0.42	0.45	0.19
*τ-Muurolol*	31.809	1645	-	0.18	0.1	0.35	0.33	0.19	0.15	0.11
***α-cadinol***	32.291	1658	5.62	1.43	1.69	1.99	1.89	1.69	1.65	1.47
*Veridiflorol*	39.353	1859	1.74	0.98	0.65	0.83	1.16	0.88	1.2	0.79
*Other compounds*										
*Acetic acid*	1.681		-	-	-	-	-	0.06	0.3	0.03
*Dihydroactinolide*	27.460	1531	1.3	0.57	0.59	0.37	0.56	0.31	0.88	0.04
*7-Methoxycoumarin*	34.725	1724	1.08	0.23	-	-	-	-	-	-
*Bisabolol oxide A*	35.575	1749	5.88	1.09	0.8	0.62	0.49	0.33	0.27	0.17
*Hexahydrofarnesyl acetone*	38.963	1847	1.07	0.31	0.28	0.33	0.32	0.4	0.64	0.4
1,2-Benzenedicarboxylic acid, bis(2-methylpropyl) ester	39.729	1870	4.8	1.04	1.45	0.53	0.49	1.29	0.5	0.22
Methyl hexadecanoate	41.658	1927	0.15	-	-	-	-	0.08	0.08	0.04

## Data Availability

The original contributions presented in this study are included in the article/Supplementary Materials. Further inquiries can be directed to the corresponding authors.

## Supplementary Materials

The following supporting information can be downloaded at: https://www.mdpi.com/article/10.3390/plants13243457/s1.
