# Supplementary material for: Unlocking the Unique Potential of Thymus pannonicus: Exploring the Efficacy of Supercritical CO2 Extraction, with and Without Pre-Treatments
Source: Plants (Basel). 2024 Dec 10;13(24):3457. doi: 10.3390/plants13243457 (PMC11676998; doi:10.3390/plants13243457)
Supplement: Supplementary file 1 [file plants-13-03457-s001.zip › plants-3323901-supplementary.pdf]

SUPPLEMENTARY FOLDER

Unlocking the Unique Potential of *Thymus pannonicus*: Exploring the Efficacy of Supercritical CO_2 Extraction, with and Without Pre-Treatments

Siniša Simić [1], Senka Vidović [1], Stela Jokić [2], Nataša Milić [3], Krunoslav Aladić [2], Zoran Maksimović [4], Jovana Drljača Lero [3] and Aleksandra Gavarić [1,*]

[1] Faculty of Technology Novi Sad, University of Novi Sad, Bulevar cara Lazara 1, 21000 Novi Sad, Serbia; sinisa.simic@uns.ac.rs (S.S.); senka.vidovic@uns.ac.rs (S.V.)
[2] Faculty of Food Technology Osijek, Josip Juraj Strossmayer University of Osijek, Franje Kuhača 18,
31000 Osijek, Croatia; stela.jokic@ptfos.hr (S.J.); k2aladic@gmail.com (K.A.)
[3] Faculty of Medicine, Department of Pharmacy, University of Novi Sad, Hajduk Veljkova 3, 21000 Novi Sad, Serbia; natasa.milic@mf.uns.ac.rs (N.M.); jovana.drljaca-lero@mf.uns.ac.rs (J.D.L.)
[4] Faculty of Pharmacy, University of Belgrade, Vojvode Stepe 450, 11221 Belgrade, Serbia; zoran.maksimovic@pharmacy.bg.ac.rs
* Correspondence: cvejina@uns.ac.rs

Total Ion Chromatogram (TIC) for all of the analyzed samples

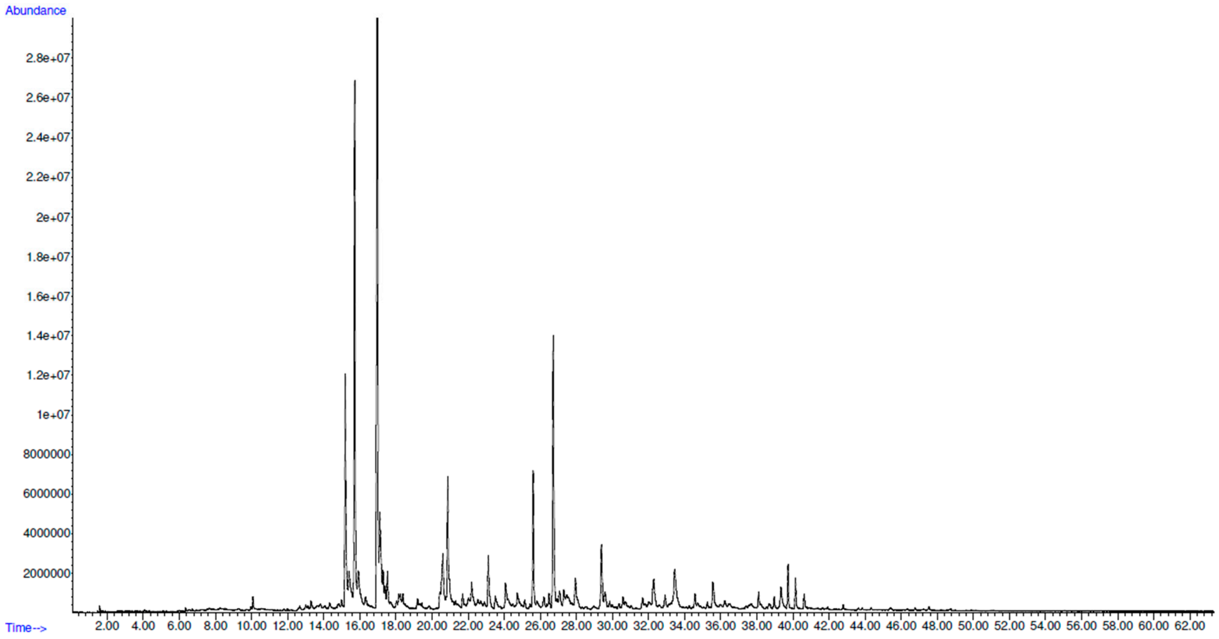

Figure 1. Total ion chromatogram (TIC) for the sample E2, with the x-axis representing time [min] and the y-axis representing signal intensity.

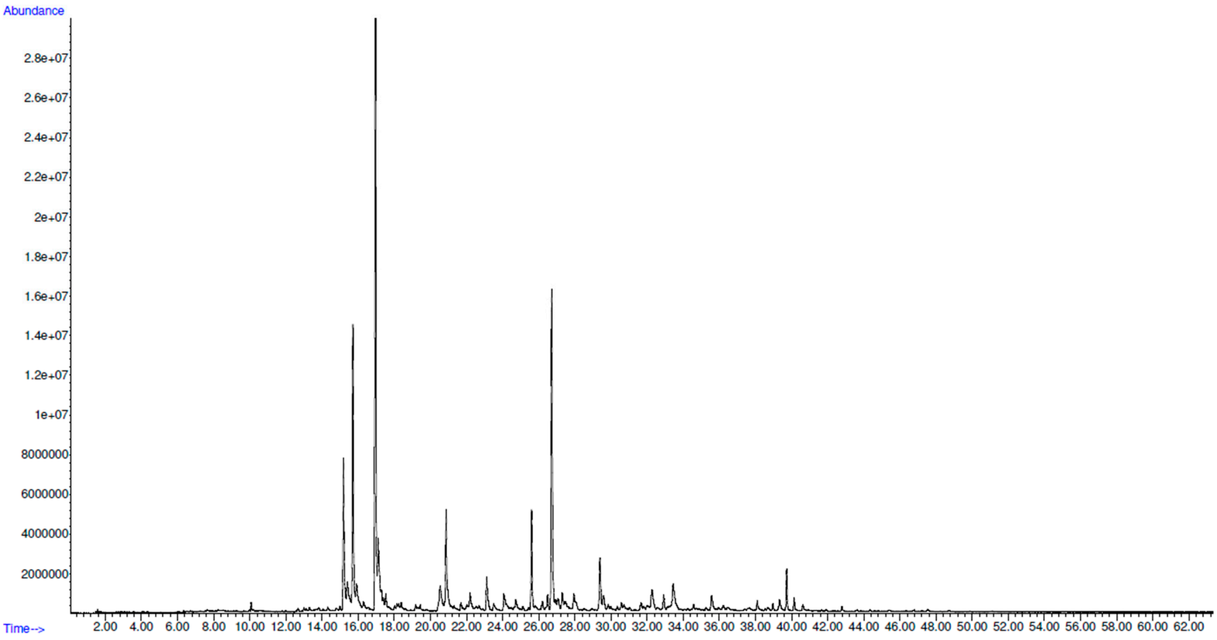

Figure 2. Total ion chromatogram (TIC) for the sample E3, with the x-axis representing time [min] and the y-axis representing signal intensity.

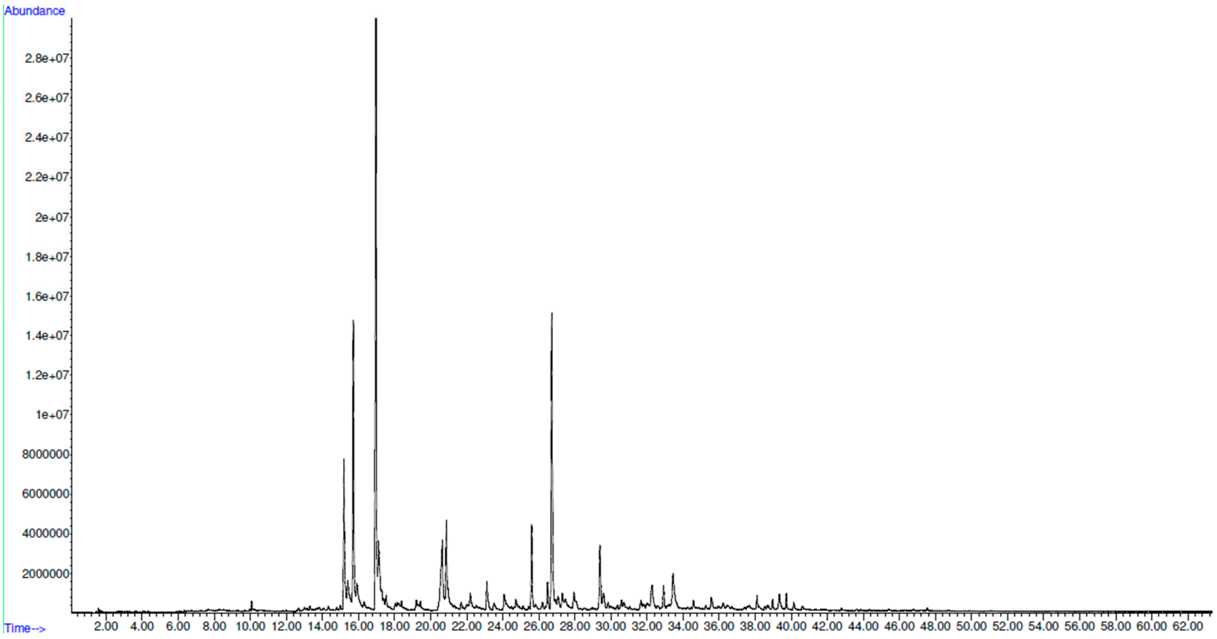

Figure 3. Total ion chromatogram (TIC) for the sample E4, with the x-axis representing time [min] and the y-axis representing signal intensity.

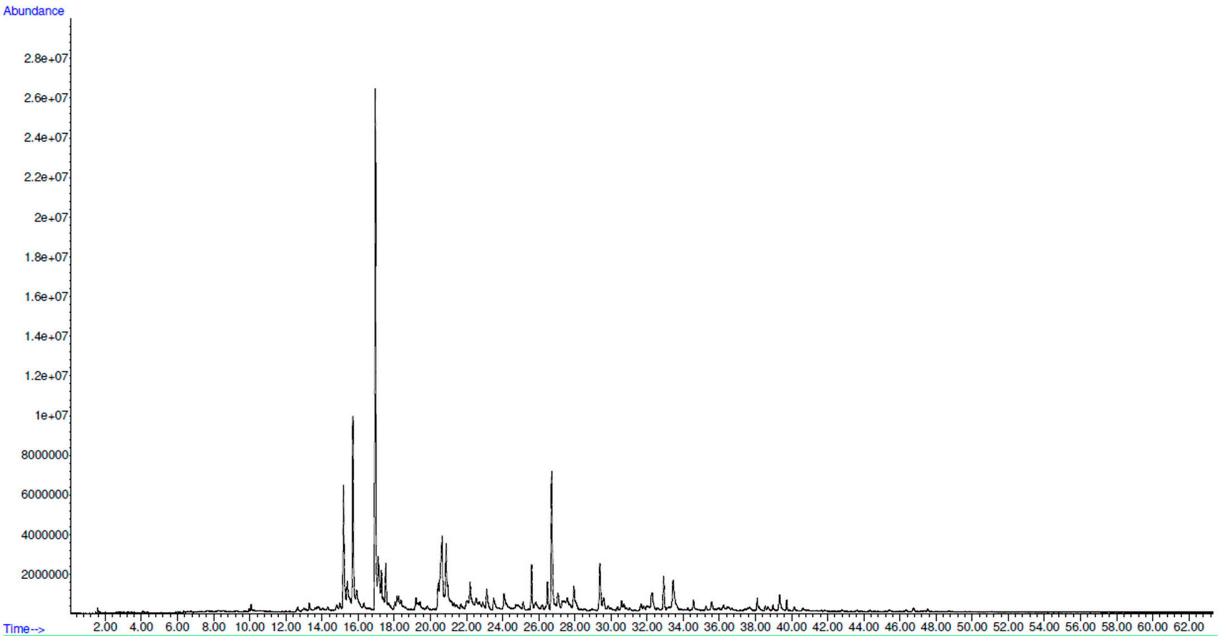

Figure 4. Total ion chromatogram (TIC) for the sample E5, with the x-axis representing time [min] and the y-axis representing signal intensity.

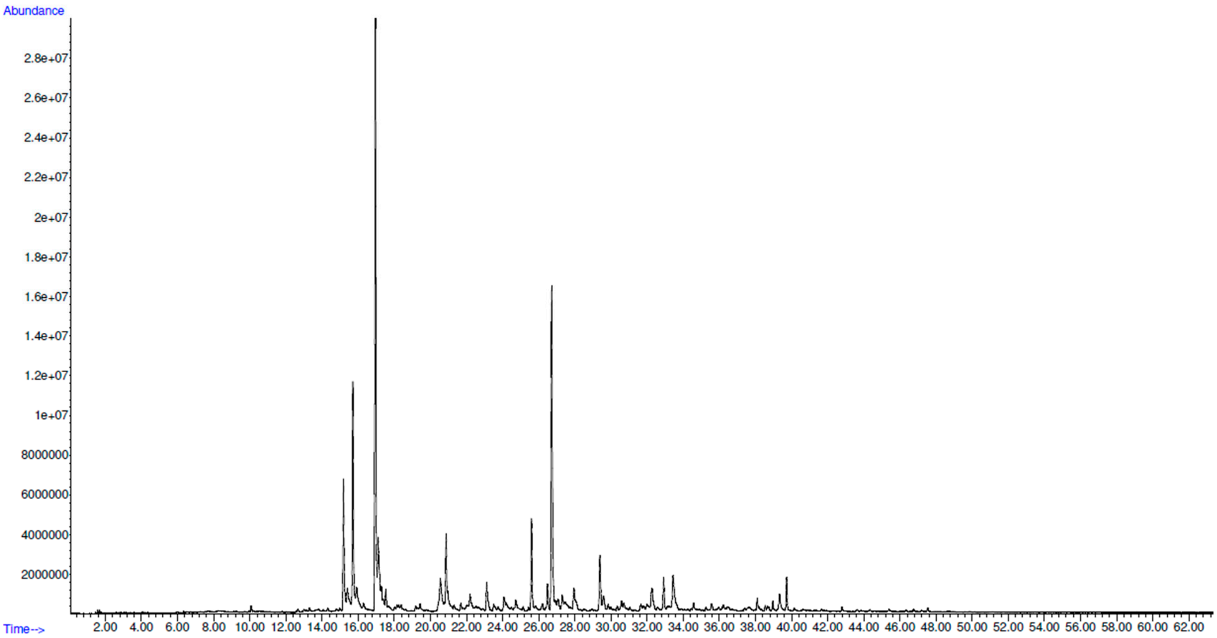

Figure 5. Total ion chromatogram (TIC) for the sample ENZ1, with the x-axis representing time [min] and the y-axis representing signal intensity.

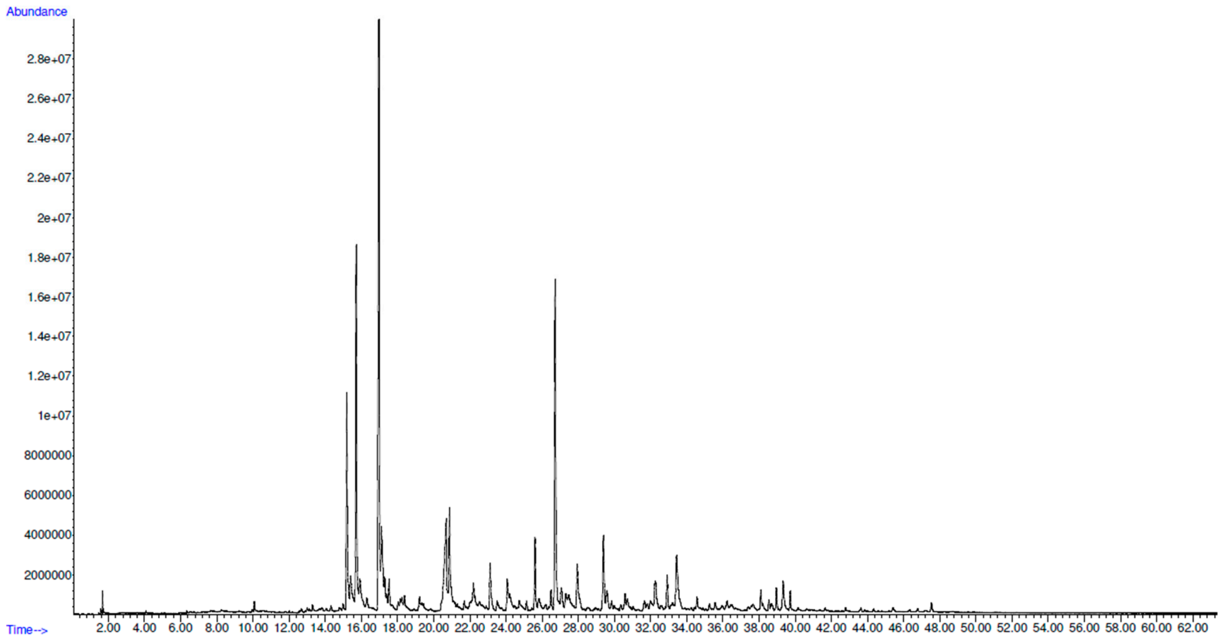

Figure 6. Total ion chromatogram (TIC) for the sample ENZ2, with the x-axis representing time [min] and the y-axis representing signal intensity.

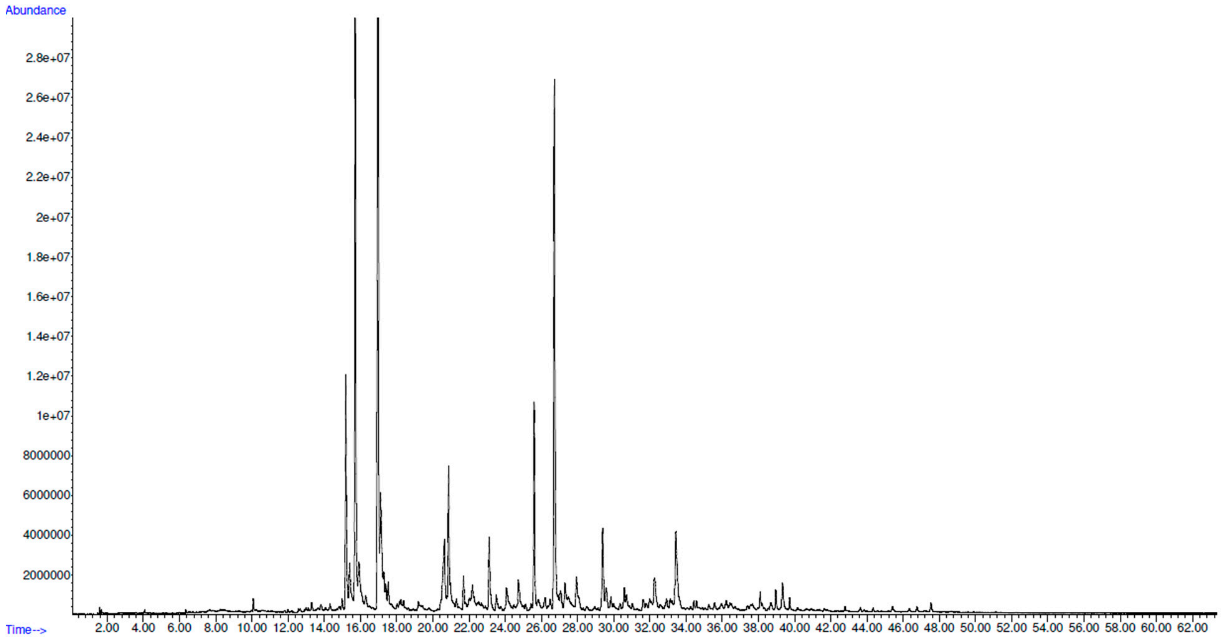

Figure 7. Total ion chromatogram (TIC) for the sample MW, with the x-axis representing time [min] and the y-axis representing signal intensity.

Mass spectra of the major components.

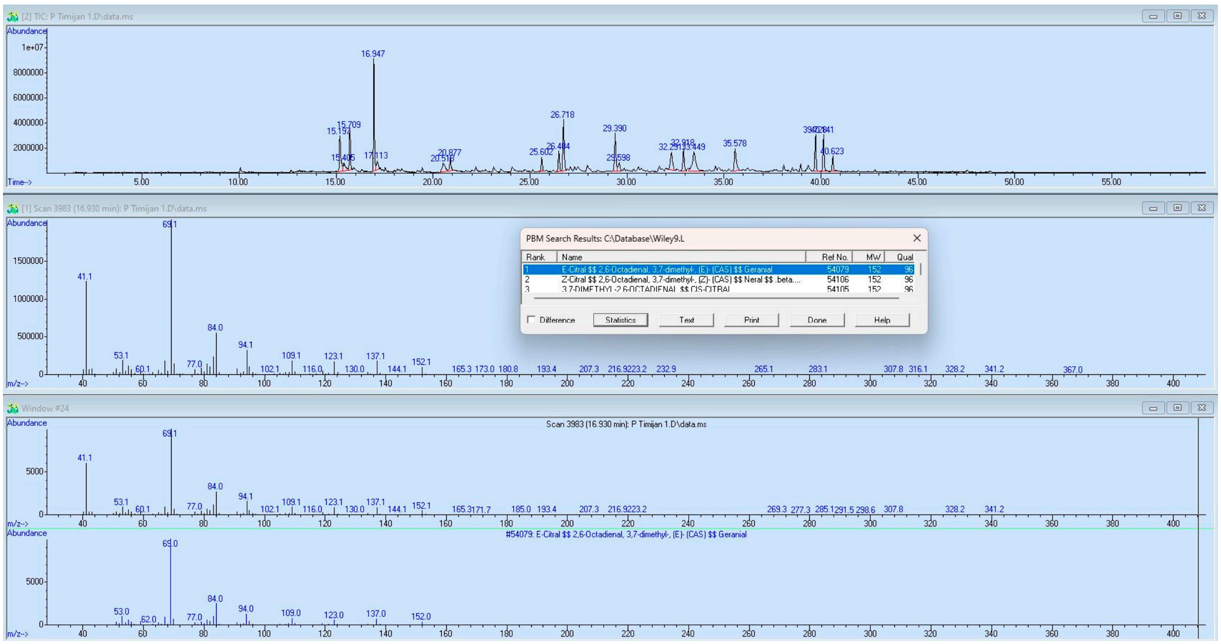

Figure 8. Mass spectra for the component (E)-citral

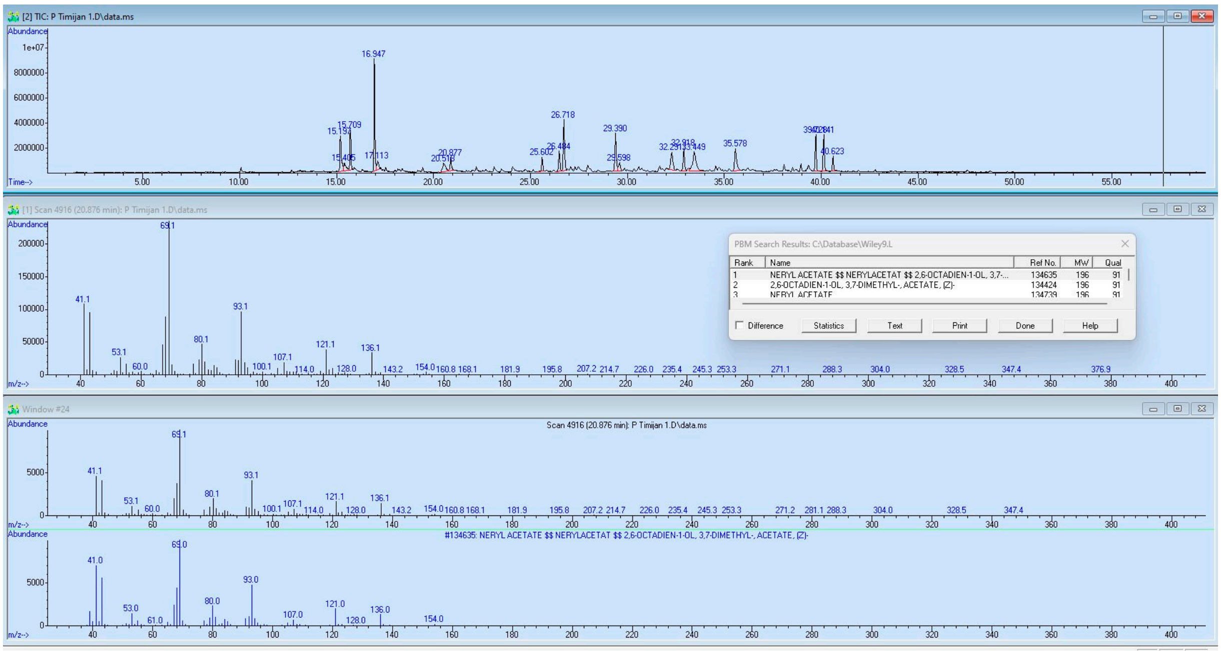

Figure 9. Mass spectra for the component Neryl acetate

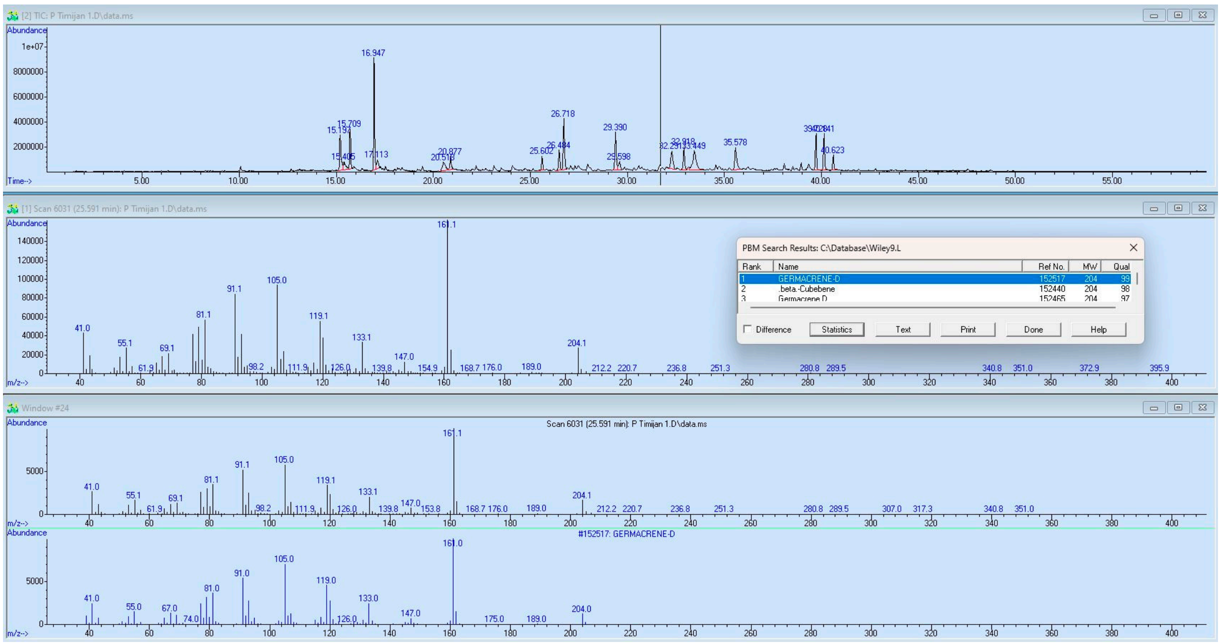

Figure 10. Mass spectra for the component Germacrene D

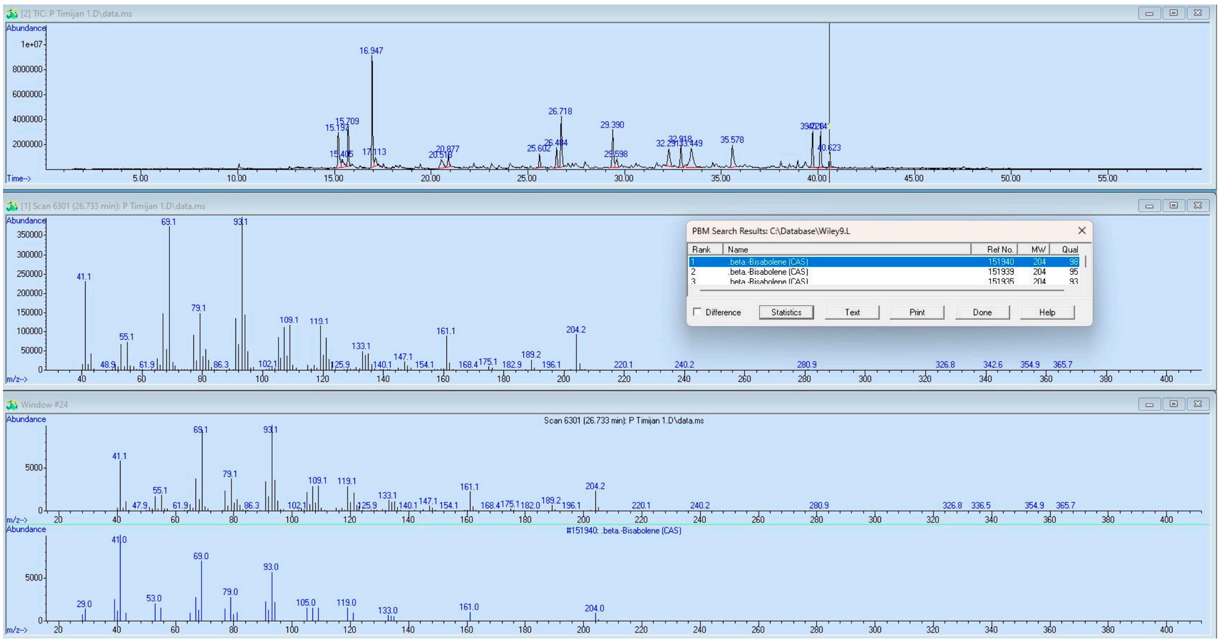

Figure 11. Mass spectra for the component β-Bisabolene

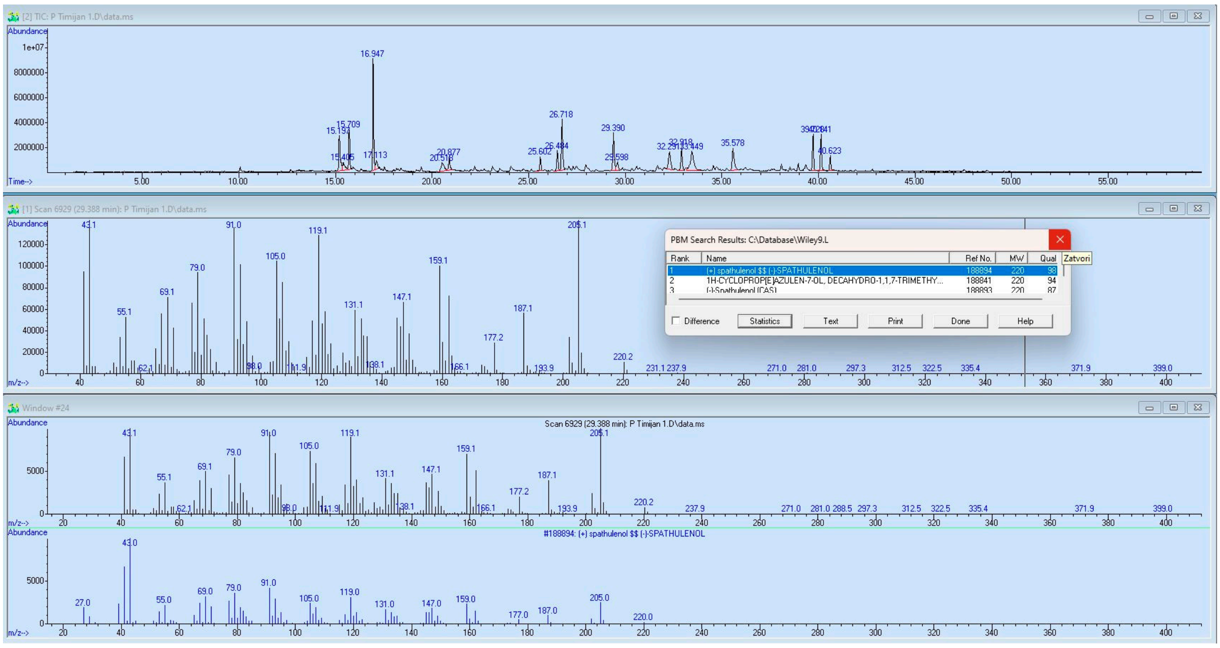

Figure 12. Mass spectra for the component Spathulenol
